# Predictors of depression among caregivers of patients with severe mental illness in Northwest Ethiopia, 2023: an explanatory sequential mixed-method study

**DOI:** 10.3389/fpsyt.2024.1422104

**Published:** 2024-09-20

**Authors:** Birhanu Mengist Munie, Zelalem Birhan, Getasew Legas, Sintayehu Asnakew, Amsalu Belete, Getnet Mihretie Beyene, Kirubel Shiferaw, Anemut Tilahun Mulu, Yohannes Tesfahun Kassie, Tigabu Munye Aytenew, Assasahegn Tedla

**Affiliations:** ^1^ Department of Psychiatry, College of Health Sciences, Debre Tabor University, Debre Tabor, Ethiopia; ^2^ Department of Psychiatry, College of Medicine and Health Sciences, Wollo University, Dessie, Ethiopia; ^3^ Department of Biochemistry, College of Health Sciences, Debre Tabor University, Debre Tabor, Ethiopia; ^4^ Department of Emergency and Critical Care Nursing, College of Health Sciences, Debre Tabor University, Debre Tabor, Ethiopia; ^5^ Department of Nursing, College of Health Sciences, Debre Tabor University, Debre Tabor, Ethiopia

**Keywords:** caregiver, depression, Ethiopia, mental illness, severe mental illness

## Abstract

**Background:**

Severe mental illness results in an enormous social and economic burden on affected individuals, their families, and communities, especially in developing countries, such as Ethiopia.

**Objective:**

The aim of this study was to assess the level of depression among caregivers of patients with severe mental illness in Debre Tabor Town, Northwest Ethiopia in 2023.

**Methods:**

This institution-based explanatory mixed study was conducted at Debre Tabor Compressive Specialized Hospitals between September 30 to October 30, 2023. A systematic random sampling technique was used to select 260 study participants, and a public health questionnaire was used to assess depression. Epicollect5 was used to collect data, which were then exported to the SPSS-25 for analysis. Variables with a *p*-value <0.25 were considered candidates for the multivariate logistic regression analysis. The odds ratios with a 95% confidence interval were used to determine the strength of the association. An in-depth interview was conducted with 11 participants, selected using purposive sampling.

**Results:**

The prevalence of depression was 31.3% (95% CI = 29.7–38.6). The multivariate analysis showed that being female (AOD = 2.43, CI = 1.42–7.23), divorced/widowed (AOD = 1.8, CI = 1.32–6.34), poor social support (AOD = 2.2, CI = 1.9–5.87), and perceived stigma (AOD = 2.33, CI = 0.24–13.22) were positively associated with depression. The qualitative results suggest that being female, illiterate, severity of the illness, poor social support, and stigma were factors for depression.

**Conclusions and recommendations:**

The prevalence of depression was high among caregivers of patients with severe mental illness. Female sex, being divorced or widowed, being illiterate, poor social support, and perceived stigma were the contributing factors. This implies that a greater focus on caregivers and the government increases mental health literacy and mental health community services.

## Introduction

According to the *Diagnostic and Statistical Manual of Mental Disorders, Fifth Edition* (*DSM-5*), the diagnosis of a major depressive episode (MDE) requires five or more symptoms to be present within a 2-week period. One of the symptoms should, at least, be either a depressed mood or anhedonia (loss of interest or pleasure). Supportive symptoms of MDE include appetite or weight changes, sleep difficulties (insomnia or hypersomnia), psychomotor agitation or retardation, fatigue or loss of energy, diminished ability to think or concentrate, feelings of worthlessness or excessive guilt, and suicidality (1).

Depression is a common psychiatric disorder and a significant public health problem with an estimated lifetime prevalence of 10% in the general population, and in clinical settings, it may reach 20%, and it is predominant in caregivers of patients with severe mental illness and chronic medical illness (2–4).

In 2008, the World Health Organization (WHO) ranked major depression as the third cause of disease burden worldwide and projected that the disease will rank first by 2030 (5).

In 2019, one in every eight people, or 970 million people around the world, was living with a mental disorder, with anxiety and depressive disorders being the most common. In 2019, 280 million people were living with depression, including 23 million children and adolescents (6).

Globally, 450 million people are estimated to be affected by mental disorders at any one time. These include 121 million people with depression, 24 million people with schizophrenia, and 37 million people with dementia (7).

Depression is the fourth most important contributor to the global burden of disease, and 4.4% of the total disability-adjusted life years (DALYs) is explained by depression (8, 9). Epidemiological evidence also shows that approximately 1.2% of the total burden in Africa to 8.9% in high-income countries is explained by depression (10). In sub-Saharan Africa (SSA), mental illness accounts for 19% of years lived with disabilities (YLDs) regionally. These estimates indicate that mental illness is one of the leading causes of ill health and disability (11).

Caregiving is a demanding and difficult task that may have a negative impact on the quality of life of caregivers. It has been reported that chronic caregiving becomes a burden for caregivers and leads to psychosocial distress and compromised quality of life in caregivers (12).

Studies have shown that caring for a mentally ill patient affects various aspects of caregivers’ lives, including their quality of life and socioeconomic status (13). For instance, family caregivers of patients with severe mental illness are usually required to provide financial support and endure the burden of economic difficulties. They also provide physical and emotional support to the patient and bear the emotional and physical stress resulting from patients’ disturbing behaviors that consequently affect daily routines and the ability to undertake the usual social activities that lead to psychological distress (14).

Severe mental illness results in an enormous social and economic burden on affected individuals, their families, and communities (15).

Caregivers experience psychological and emotional distress, reduction in social contact, and financial difficulties, and report lower life satisfaction and poor physical and mental health as a result of caregiving; this chronic stress and daily hassles cause profound objective and subjective burden for caregivers of relatives with severe mental illness (16, 17).

Caring for patients with severe mental illness demands a considerable amount of time and other resources from caregivers, who suffer twice as much as the general population (18).

However, stigma not only impacts the individual experiencing the severe mental illness but also affects those they are closely associated with (e.g., family members and primary caregivers), which directly or indirectly leads the individual to depression (19).

The prevalence of depression among caregivers of patients with severe mental illness is greater than that among the general population because of the burden of providing care to these patients with severe mental disease.

Studies in Ethiopia reported that among caregivers of patients with severe mental illness, the prevalence of depression was 19% (20) and 57.7% (21); this gap indicates the need for further investigation and focus on caregivers of patients with severe mental illness. Another study had a depression prevalence of 56.7%, which indicates that there is a high prevalence of mental distress among those who care for people with severe mental illness (22).

Depression among caregivers of patients with mental illness has been associated with many factors such as patient age, employment, income, ethnicity, educational level, perceived stigma, poor social support, and prolonged stays with the patient (23–25).

On the other hand, the patient’s condition, caregiving burden, duration of caregiving, spouse caregiver, caregiver being unemployed, caregiver with chronic disease, caregiver’s sleep quality, caregiver’s avoidance, financial problems, and female sex were positively associated with depression, whereas the overall quality of life of the caregiver, pre-loss grief, caregiver’s education level, caregiver’s age, caregiver’s sense of coherence, and caregiver’s bond with patient were negatively associated with depression in caregivers of severe mental illness and among primary caregivers of severe mental illness who are female, mother, gave care for greater than five years, have no other caregiver (21, 26).

The impact of caregiving on the physical and mental health of caregivers is well documented. When compared to non-caregivers, caregivers experienced higher levels of depression, increased stress, more outpatient visits, and a lower quality of life (27).

Studies have shown that the status of caregivers of patients with mental disorders has been neglected in some countries, especially developing countries, including Ethiopia. Although some of the needs and challenges for caregivers and family members of patients may be common, they have unique needs and many uncertainties (28). However, many health professionals and healthcare providers, particularly psychiatric nurses, often focus their care on the patient and ignore the family and main caregivers of the patient. These professionals exclude them from the disease, treatment, and decision-making processes and do not consider their needs; hence, families do not have a chance to express their concerns and needs, and are at risk of serious psychological problems (29).

By identifying the problems and challenges of caregivers of patients admitted to the hospital, psychiatric nurses can plan their issues and problems.

Although numerous studies have been conducted on family caregivers of patients with mental illnesses in Ethiopia, a comprehensive exploration of their challenges has not been conducted. Therefore, mixed study evidence and other relevant evidence were explored to capture the challenges faced by caregivers of patients with mental health problems in the Ethiopian context and to reveal the problems of the caregivers. This study aimed to assess the predictors of depression and its associated factors among the primary caregivers of patients with mental illness.

## Methods and materials

### Study area and period

The study was conducted from 30 September to 30 October 2023 in Debre Tabor Comprehensive Specialized Hospital (DTCSH), Debre Tabor city, South Gondar zone, and Amhara region, which is located in the northwest part of Ethiopia. Debre Tabor Town is the capital city of the South Gondar zone, which is 666 km from Addis Ababa and 99 km from Bahir Dar (the capital city of the Amhara region). According to the 2007 population census report, the total population of South Gondar is estimated to be 2,051,738, and the zone has seven primary hospitals and one comprehensive specialized hospital.

The DTCSH has 30 inpatient beds; four outpatient department rooms and one substance rehabilitation unit are available in the psychiatry unit, and there are seven mental health specialists (MSc in integrated clinical and community mental health) and four BSc psychiatric nursing staff. The psychiatry section serves a total patient population of 7,000 persons per year, with approximately 530 patients with severe mental illness visiting their caregivers on a monthly basis.

### Study design

An institutional-based explanatory sequential mixed study design was used.

### Population

Source population: All caregivers of patients with severe mental illness in DTCSH.

Study population: All caregivers of patients with severe mental illness in DTCSH during the data collection period.

### Eligibility criteria

#### Inclusion criteria

All adult caregivers of patients with severe mental illness at the DTCSH during the study period were included in the study.

#### Exclusion criteria

Caregivers who were unable to provide proper information (unconscious, severely ill, or unable to communicate) during the study period were excluded.

Caregivers who provided care for less than 6 months during the study period were likewise excluded.

### Sample size determination

The sample size was determined by using a single population proportion formula by considering the rate of depressive disorders among caregivers of patients with severe mental illness to be 19%, as it was reported by a study conducted in Southwest Ethiopia (20) with 5% marginal error (d) and 95% confidence interval of certainty (α = 0.05).


$$\text{n}=\;\frac{({\text{z}}_{\text{α}/2)}^{\;2}\;\text{p}\left(1-\text{p}\right)}{{\text{d}}^{2}}$$

$n\;=\;\frac{{\left(1.96\right)}^{2}\;0.19*\left(1-0.19\right)}{{\left(0.05\right)}^{2}}$ = 237 for a possible non-response rate addition of 10%, which was the final sample size of 260. Eleven participants were purposively selected for the qualitative analysis.

### Sampling procedure

For quantitative analysis, systematic random sampling was used to select study participants. The psychiatry clinic provides services to an average of 530 patients with SMI who visit their caregivers at the DTCSH per month. The sampling interval (*K*) was determined by dividing the expected number of caregivers of patients with SMI per month (530) by the sample size (260), which provided an approximate sampling interval of 2. Then, data were collected from each study participant with an interval of two until the desired sample size was reached, and the starting point was selected using the lottery method. For Part A, 11 study participants were selected using purposive sampling until information saturation was reached.

### Study variables

Depression (yes or no) was used as the outcome variable. The independent variables included sociodemographic factors (age, sex, residence, educational status, occupational status, income, and marital status), psychosocial factors (duration of caregiving, social support, and stigma), clinical factors (having known medical illness and having a family history of mental illness), substance-related factors (alcohol use, khat use, and tobacco use), and patient-related factors (age, sex, educational status, comorbid medical illness, type of diagnosis, and severity of illness).

### Operational definitions

Depression: Depression was measured as a PHQ-9 score > 10 on the depression scale (30).

Ever substance use: Those who had used substances in their lifetime (31).

Current substance use: Those who had used substances within the last 3 months (31).

Severe mental illness: The diagnosis of schizophrenia, schizoaffective disorder, bipolar disorder, or major depressive disorder is thought to cause major morbidity and mortality (32).

Quality of life: Caregivers with scores less than 50 were categorized as having poor quality of life (33).

Caregiver: A family member/relative/any person who has the most frequent contact with the patient; who provides unpaid support to the patient financially, socially, psychologically, and physically; and who has mostly been vital in the patient’s treatment visit.

### Data collection procedure and instruments

Data were collected via the Epicollect5 software application on an Android phone and then uploaded to the creator. Four BSc psychiatric professional personnel from the study location and two supervisors collected data via face-to-face interviews. Subsequently, caregivers who met the eligibility criteria were given an informed consent form to sign after being informed about the study’s goals, objectives, and purpose. The data collectors carry out the interview of qualified and willing caregivers of SMI patients at a convenient location while supervisors monitor the data collection procedure.

A semi-structured sociodemographic interviewer-administered questionnaire was used to obtain data such as age, sex, ethnicity, marital status, educational attainment, employment status, income, residence, type of diagnosis, duration of caregiving, and medical history of the patient and the caregiver.

A structured questionnaire, the Public Health Questionnaire (PHQ-9), was used to assess depression among primary caregivers. The PHQ-9 scores range from 0 to 27. Each of the nine items is scored from 0 (“not at all”) to 3 (“nearly every day”). A PHQ-9 score of 10–14 indicates moderate depression and 15–19 indicates moderately severe depression. A score of 20–27 on PHQ-9 indicates severe type of depression that requires immediate initiation of therapy (30, 34). Moreover, PHQ-9 has been validated in an Ethiopian healthcare context, with a specificity and sensitivity of 67% and 86%, respectively. A cutoff point of ≥10 was used to screen for depression in this study (30).

Social support scale: Social support was measured using the Oslo Social Support Scale (OSSS-3) (35). The OSSS-3 total score ranged from 3 to 14. Scores from 3 to 8 indicate poor support, scores from 9 to 11 indicate intermediate support, and scores between 12 and 14 indicate strong social support. It has an acceptable internal consistency (α = 0.640) and has been used in Ethiopian settings (36–38).

Severity of illness: The CGI severity scale responses of 1–3 are taken as mild, 4 as moderate, and 5–7 as severe illness for both subjective and objective severity assessments (39, 40).

Stigma: The Family Interview Schedule (FIS) questionnaire, which was developed as part of the World Health Organization, had good internal consistency (Cronbach’s α = 0.92). The FIS includes 14 questions on families’ experiences of stigma in the community. Each stigma item was rated on a four-point scale, not at all (0), sometimes (1), often (2), and many (3) with respect to stigma. To assess the distribution of stigma responses between groups, a stigma sum score was computed by summarizing all positive responses (≥1) for each of the 14 items. The presence of only one positive answer in the stigma questionnaire was sufficient to represent a form of perceived stigma (41).

Quality of life: The WHOQOL-BREF questionnaire is a 26-item, five-point Likert scale developed by the WHO to assess quality of life over the past 2 weeks in four domains (42–45).

Finally, the English version of the questionnaire was translated into Amharic (the local language) for easier comprehension by data collectors and respondents and then translated back into English by another individual to ensure semantic comparability.

Qualitative part: Data were gathered using an in-depth interview method. An interview guide was used as a data-gathering tool, which consisted of open-ended, semi-structured questions that could extract information in terms of the research aims and was created by the researcher after reviewing several studies. An audio tape record was used to avoid distraction from extensive note-taking. The interviews ranged from 30 to 90 min in length, using a tape recorder. Each individual was briefed about the study by the principal investigator, and the interviews were arranged between the principal investigator and the interviewee.

### Data quality control

The questionnaire was first written in English, and then translated into Amharic by language experts for data-gathering purposes, and finally translated back to English to verify consistency. The quality of data was ensured through training for 1 day before data collection for data collectors and regular supervision, immediate feedback, and reviewing each of the completed questionnaires daily by the principal investigator.

### Data processing, analysis, and interpretation

Data were checked for completeness, entered into the Epicollect5 software, downloaded to Microsoft Excel, and exported to SPSS-25. Descriptive statistics, such as mean, standard deviation, proportions, frequency, cross-tabulations, and percentages, were used to describe the dependent and independent variables in the study. The results are presented as charts, graphs, and tables. Bivariate and multivariate logistic regression analyses were used to identify depression and its related factors. Variables that were significant in the binary analysis (*p* ≤ 0.25) were considered in the multivariable logistic regression analysis. Its strength is presented using odds ratios and 95% confidence intervals. The results are presented as words, tables, and figures.

Qualitative part: Data were obtained using in-depth interview and by transcribing the information to paper and grouping similar ideas, and then open code 4.03 software was used for thematic analysis.

## Results

### Sociodemographic characteristics of the respondents

A total of 260 participants participated in this study, with a response rate of 100%. The mean age of the respondents was 36.8 years with an SD of ±11.02 years, and the majority were between the ages of 30 and 40 years; 154 (59.3%) resided in urban areas. The majority of the respondents were men (139, 53.5%), married (180, 69.2%), Amhara by ethnicity (255, 98.1%), orthodox followers (187, 71.9%), and had an average monthly income of $78.2 (**Table 1**).

**Table 1:** Sociodemographic characteristics of study participants among caregivers of patients with severe mental illness at Debre Tabor, Northwest Ethiopia, 2023 (quantitative part, *n* = 260).

Characteristic	Category	Frequency	Percentage
Sex	Male	139	53.5
	Female	121	46.5
Age	20–30	75	28.8
	30–40	80	30.8
	40–50	69	26.6
	≥50	36	13.8
Residence	Rural	106	40.8
	Urban	154	59.2
Marital status	Married	180	69.2
	Single	50	19.2
	Divorced/widowed	30	11.6
Religion	Orthodox	187	71.9
	Muslim	57	21.9
	Protestant	14	5.4
	Other	2	0.8
Ethnicity	Amhara	255	98.1
	Oromo	4	1.5
	Tigrawi	1	0.4
Educational status	Cannot read and write	68	26.2
	Primary education	79	30.3
	Secondary education	46	17.7
	College diploma	25	9.6
	Degree and above	42	16.2
Job	Governmental employee	75	28.8
	Farmer	106	40.8
	Merchant	54	20.8
	Daily labor	6	2.3
	Job less	19	7.3
Estimated monthly income	Poor income (<$51.2)	67	25.8
	Medium income ($51.2–$87.2)	114	43.8
	Good income (>$87.2)	79	30.4
Kinship with the patients	Parents	114	43.8
	Spouse	56	21.5
	Children	35	13.5
	Sister/brother	43	16.5
	Other	12	4.6

### Qualitative part

Eleven primary caregivers were selected, consented to participate, and interviewed. Most of the caregivers (7/11, 66.6%) were men, and 80% were between 30 and 45 years old. More than 90% (10/11) of the caregivers had diplomas, and above the majority had secured occupations (**Table 2**).

**Table 2:** Sociodemographic characteristics of study participants among caregivers of patients with severe mental illness at Debre Tabor, Northwest Ethiopia, 2023 (qualitative part, *n* = 11).

Characteristic	Category	Frequency	Percentage
Sex	Male	7	66.6
	Female	4	33.4
Age	<30	1	10
	30–45 years	10	90
Residence	Rural	2	20
	Urban	9	80
Marital status	Married	9	80
	Single	0	0
	Divorced/widowed	2	20
Religion	Orthodox	11	100
Ethnicity	Amhara	11	100
Educational status	Primary education	1	10
	College diploma and above	10	90
Job	Governmental employee	8	70
	Farmer	1	10
	Merchant	2	20
Estimated monthly income	Poor income (<2,981)	1	10
	Medium income (2,982–5,020)	8	70
	Good income (>5,021)	2	20
Kinship with the patients	Parents	6	50
	Spouse	2	20
	Children	2	20
	Sister/brother	1	10

### Patients-related characteristics

In addition to the study participants, the study investigated the patients’ related characteristics; 150 (57.7%) participants were women, with a mean age of 35.67 years with an SD of ±11.845; 117 (45%) were single, and in terms of educational status, 97 (37.3%) attended primary education. Nearly half (123, 47.3%) of the patients were diagnosed with schizophrenia spectrum disorder with a mean duration of illness of 3.459 ± 2.19 years. A total of 51 (19.3%) patients had additional comorbid medical illnesses (**Table 3**).

**Table 3:** Patient-related characteristics of caregivers of patients with severe mental illness at Debre Tabor, Northwest Ethiopia, 2023 (*n* = 260).

Variables	Categories	Frequency	Percentage
Sex	Female	150	57.7
	Male	110	42.3
Age	<30	127	48.8
	30–40	77	29.8
	>50	56	21.4
Educational status	Cannot write and read	58	22.3
	Primary education	97	37.3
	Secondary education	65	25
	Diploma graduate	13	5.0
	Degree and above	27	10.4
Marital status	Single	117	45
	Married	95	36.5
	Divorced/widowed	48	18.5
Type of diagnosis	Schizophrenia spectrum disorder	123	47.3
	Bipolar-related disorder	87	33.5
	Major depression-related disorder	50	19.2
Duration of caregiving	6 months up to 1 year	124	47.7
	1–5 years	101	38.8
	>5 years	35	13.5
Comorbidity of medical illness	Yes	52	20.0
	No	208	80.0

### Clinical, psychosocial, and substance characteristics of the study participants

A total of 33 (12.7%) caregivers had a history of mental illness, whereas 26 (10%) of the study participants had a chronic medical condition. Almost one-fourth of the individuals reported using substances within the previous 3 months, and nearly half reported having no social support. A total of 99 (38.1) participants had a poor quality of life, and 155 (59.6) participants were stigmatized (**Table 4**).

**Table 4:** Clinical, psychosocial, and substance characteristics of caregivers of patients with severe mental illness at Debre Tabor, Northwest Ethiopia, 2023 (*n* = 260).

Variables	Categories	Frequency	Percentage
Chronic medical illness	Yes	26	10
	No	234	90
History of mental illness	Yes	55	21.2
	No	205	78.8
Social support	Strong social support	69	26.4
	Intermediate social support	67	25.8
	Poor social support	124	47.7
Ever used of substance	Yes	90	34.6
	No	170	65.4
Current use of substance	Yes	59	22.7
	No	201	77.3
Severity of the illness of the patients	Mild	140	53.8
	Moderate	69	26.4
	Severe	51	19.6
Perceived stigma	Yes	155	59.6
	No	105	40.4
Quality of life	Good	161	61.9
	Poor	99	38.1

### Prevalence of depression among caregivers of patients with severe mental illness

The overall prevalence of depression among primary caregivers of patients with severe mental illness was 33.1% (86).

### Factors associated with depression

In the binary analysis of depression in relation to each explanatory variable, female sex, marital status, educational status, occupation, kinship with the patient, type of diagnosis, duration of providing care, chronic medical illness, poor social support, perceived stigma, and poor quality of life were found to be significant at a *p*-value of less than 0.25.

These factors were entered into multivariable binary logistic regression analysis to control for confounding effects. In the multivariate analysis, being female, being divorced or widowed, being unable to read and write and to attend primary education, providing care for patients with schizophrenia spectrum disorder and bipolar disorder, giving care for more than 5 years, having chronic medical illness, providing care for severely ill patients, having poor social support, and being stigmatized were significantly associated with depression (*p* < 0.05).

Female caregivers were 2.43 times more likely to develop depression than male caregivers (AOR: 2.43, CI = 1.42–7.23). The odds of developing depression were 1.8 times higher among respondents who were divorced or widowed than among married respondents (AOR: 1.8, CI = 1.32–9.90).

The likelihood of developing depression was 3.12 times higher among respondents who could not read and write (AOR: 3.12, CI = 1.22–9.90) and 1.93 times higher among respondents who attended primary school (AOR: 1.93, CI = 1.02–4.97) than among those who had a degree and above.

Being a caregiver of patients with schizophrenia spectrum disorders was 2.8 times more likely to develop depression than caregivers of patients with depression-related disorders (AOR: 2.8, CI = 1.09–7.55). Being a caregiver of patients with bipolar and related disorders was 1.69 times more likely to develop depression than being a caregiver of patients with depression-related disorders (AOR: 1.69, CI = 1.07–5.52).

The odds of developing depression were 1.98 times higher among those respondents who were giving care for more than 5 years than those who were giving care for less than 1 year (AOR: 1.98, CI = 1.05–5.56).

The likelihood of developing depression was 2.35 times more likely among respondents who had medical illnesses than among those who had no medical illnesses (AOR: 2.35, CI = 1.75–13.7). The odds of developing depression were 2.20 times higher among participants with poor social support than among those with strong social support (AOR: 2.20, CI = 1.90–5.87). The odds of developing depression were 2.33 times higher among participants who had perceived stigma than among those who did not perceive stigma (AOR: 2.33, CI = 0.24–13.22) (**Table 5**).

**Table 5:** Bivariable and multivariable independent factors of depression among caregivers of patients with severe mental illness at Debre Tabor, Northwest Ethiopia, 2023 (*n* = 260).

Variable	Category	Depression		COR (95% CI)	AOD (95% CI)	*p*-value
		Yes	No			
Sex	Male	45	94	1.00	1.00	
	Female	47	74	2.38 (0.43–2.83)	2.43 (1.42–7.23)	0.003*
Marital status of the caregiver	Married	61	119	1.00	1.00	
	Single	20	30	1.41 (0.43–2.94)	1.23 (1.06–3.23)	0.26
	Divorced/widowed	11	19	1.21 (0.39 - 1.96)	1.8 (1.32–6.34)	0.001**
Educational status	Cannot read and write	30	38	3.56 (0.17–4.76)	3.12 (1.22–9.90)	0.07*
	Primary education	20	59	1.78 (1.02–2.90)	1.93 (1.02–4,97)	0.001**
	Secondary education	16	30	1.23 (1.16–2.56)	1.82 (0.82–6.04)	0.036
	College diploma	7	18	-0.14 (0.80–0.96)	1.43 (1.20, 4.92)	0.76
	Degree and above	13	28	1.00	1.00	
Occupational status	Government employee	20	55	1.00	1.00	
	Farmer	40	66	2.78 (1.79–4.67)	2.51 (1.54–5.67)	0.27
	Merchant	13	41	1.70 (1.02–3.21)	2.21 (1.05–4.21)	0.45
	Daily labor	4	2	2.50 (1.90–4.20)	2.30 (1.71–5.89)	0.67
	Jobless	9	10	3.56 (1.50–7.07)	3.87 (1.60–9.32)	0.75
Kinship with the patient	Parents	39	75	1.00	1.00	
	Spouse	16	40	2.66 (0.54–12.50)	2.36 (0.83, 4.24)	0.881
	Children	16	16	2.01 (0.44–10.15)	1.59 (0.30, 2.14)	0.535
	Sister/brother	13	30	4.21 (0.86–8.08)	2.01 (0.46, 2.99)	0.457
	Other	2	10	2.16 (0.42–11.30)	2.23 (1.87–12.45)	0.612
Type of diagnosis of the patient	Schizophrenia spectrum disorder	42	81	2.21 (0.62–2.46)	2.8 (1.09–7.55)	0.001**
	Bipolar-related disorder	29	58	1.76 (0.55–2.52)	1.69 (1.07–5.52)	**0**.024*
	Depression-related disorder	15	35	1.00	1.00	
Duration of giving care	6 months up to 1 year	35	89	1.00	1.00	
	1–5 years	35	66	1.26 (0.54–2.82)	1.33 (0.76–4.43)	0.256
	>5 years	15	20	1.33 (0.57–3.07)	1.98 (1.05–5.56)	0.001**
Medical illness	Yes	10	16	2.78 (1.43–14.30)	2.35 (1.75–13.7)	0.015*
	No	76	158	1.00	1.00	
Severity of illness	Mild	39	101	1.00	1.00	
	Moderate	26	43	1.50 (0.28–4.07)	1.87 (1.21–3.75)	0.456
	Severe	21	30	1.80 (0.42–6.81)	1.6 (1.02–7.45)	0.28
Social support	Poor	47	77	2.09 (1.57–3.98)	2.2 (1.9–5.87)	0.001**
	Intermediate	20	47	1.85 (1.41–2.75)	1.5 (1.27–5.75)	0.457
	Strong	13	56	1.00	1.00	
Quality of life	Good	51	110	1.00	1.00	
	Poor	35	64	1.18 (0.69–4.02)	1.42 (0.87–3.90)	0.489
Perceived stigma	Yes	74	81	2.82 (0.48–11.32)	2.33 (0.24–13.22)	0.001**
	No	22	83	1.00	1.00	

*p < 0.05; **p < 0.01; Hosmer–Lemeshow test, 0.55.

#### Qualitative findings

To explore the prevalence of depression among caregivers of patients with severe mental illness, in-depth interviews were conducted with participants. In addition to the interview guide, preliminary quantitative results were used to frame the discussions. Throughout the analysis, three themes focused on sociodemographic characteristics and clinical and psychosocial themes.

#### Sociodemographic characteristic theme

Female caregivers were heavily burdened because they spent most of their time in the house and because they were physically weak. One participant stated: “It is very surprising that especially if a woman, we are subjected to a lot of pressure, that is, trying to sexual harassment, fights, and insults, punishment and saying that they will not go to health services, refuse to take the medications, refuse to eat and unable to care his personal hygiene I witness while her father asks he easily respond in every activity*”* (IDI008/42/F).

In addition, being illiterate also increased burden and psychosocial distress as one participant explained: “Not learning enough leads me a lot of problems For instance, I belief that the cause for the illness was evil spirit, magical ideas, spells, cruses and sometimes it’s may be genetics due to this I was on traditional treatment (holy water, in magic house, Quran maskerat) for a long period of times due to the delay in visiting modern treatments the illness of my husband’s illness is becoming more complicated, due to my poor knowledge I was discontinue for the follow up visit two times” (IDI 005/39/F).

#### Clinical themes

Caregivers described the significant challenges associated with their care recipients’ severity of symptoms and behaviors related to physical problems.

“He is wary of people and feels as though they are always observing him. Every time we go out in public together, that is really annoying. He senses that he’s being followed by people in helicopters. We therefore frequently have to go home before nightfall because there is a lot of air activity during the night” (IDI 007**/**27/F).

“I think too much … and I cannot fall asleep after that in the morning I suffer with headache, fatigability … which is affecting my health and I acquire depression and I was treated in last year due to over stressed about my mom illness” (IDI 004/31/M).

#### Psychosocial themes

Social interaction and participation in events were challenging for caregivers of individuals with SMI, both in the family and in the community. According to reports, a person’s hostile behavior, lack of time for social gatherings, inability to perform expected social duties, and failure of children to establish their own ties outside the family, while parents failed to maintain their social relationships and may separate or become divorced, were the main causes of these challenges.

“Our social event participation is not the same to others. We used to take part in these events including neighbors’ coffee ceremony, weeding … Now we do not take part in such events because we cannot do our part, she used to make coffee in our turn … not only surprisingly if I want help from the neighbors no one help me during emergency situations because of they are fear of her” (IDI00639/M).

Caregivers also reported many psychosocial problems, such as loneliness and sadness, while observing a family member in the street shouting and exhibiting odd behavior, and the fear of developing the same illness triggered such feelings. The level of stress was reported to be especially high when the patient ran away, when the patient had to be physically restrained, and during relapse. Stress and hopelessness made them wish that the individual with the illness would die, and some tried to harm themselves.

“I think we go out a little less because he is so hard to get out of the house … So we don’t go out as much as I would like to. And we certainly don’t go to as many public places that I would like to. I used to really enjoy going out and having friends. But it just became such an issue because when I got home, he didn’t understand where I was and he would get so paranoid’ (IDI 0011/47/M).

“I have no friends. My mom has no friends. I’ve always felt stigmatized … I was always ashamed, I never wanted anybody to know [mom had schizophrenia]. As a result, I had very little friends growing up because even if I tried to establish relationships, my mom would do something ‘crazy,’ then they would no longer want to be my friend” (Daughter of a woman with bipolar disorder) (IDI002/25/M).

## Discussion

This study aimed to assess the level of depression among caregivers of patients with severe mental illness in Debre Tabor, Northwest Ethiopia. The results showed that the prevalence of depression among caregivers of individuals with severe mental illness was 33.1%, and different factors like sociodemographic (sex and educational status), clinical (severity of the illness), and psychosocial (social support and stigma) factors were explored by the study participants.

The results of this study are consistent with those studies conducted in Egypt, which were 34.1% (46) and 35.7% (47).

On the contrary, this result was lower than the study conducted in Ethiopia 56.7% (47) the variation may be in differences in the study participants in the previous study they were incorporate only child caregivers so as the fact giving children with mental illness increase the burden of the caregivers, Ghana 66.2% (47) there is tool variation they use the Beck Depression Inventory (BDI) to measured depression among the caregivers and also had study population differences, Kenya 56.2% (47) this variation may be differences in study population, sample size and socio-cultural difference and in China 53.5% (47) the variation may be there is better social support than China and they used Center for Epidemiologic Studies Depression Scale (CES-D 10) to assess depression.

In contrast, the result was higher than the study conducted in Ethiopia (19%) (48); the possible reason for the difference may be the current relentless continuation of intense conflict and war in the study area, which may magnify the prevalence of the depression among caregivers in India (28.5%); this might be due to the difference in the screening tools: the current study used PHQ-9, but they used the Montgomery–Asberg Depression Rating Scale (MADRS) in India; in Saudi Arabia (18.3%) (24), the discrepancy could be attributed to the socio-cultural differences and the study participants.

Regarding associated factors for depression, this study showed that female caregivers were 2.43 times more likely to develop depression than male caregivers; this finding is in agreement with the study conducted in China (21). In Ethiopia (49), this may be attributed to the fact that women are more at risk for sexual violence or to poor copying mechanisms, low self-esteem, and sex hormonal differences, and the fact that women spend more time in providing care and carrying out personal-care tasks more often than men; time-intensive care among women is also more likely in societies and cultures that endorse the traditional value of women as the natural caregiver of patients (50–52). This finding is supported by the qualitative findings of the participants: “I witness that while my husband orders something he easily agreed but while I try to communicate and give his medication he refuse and insult me too …” (IDI 008, caregiver of a schizophrenic patient).

The results of this study revealed that being divorced or widowed is 1.8 times more likely to develop depression among married respondents (AOR: 1.8, CI, 1.32–9.90); this finding was supported by a study conducted in Ghana (53), which included divorce/widowed participants. Separation was associated with increased feelings of anxiety and loneliness, and no one shared the burden of care of the patients and increased risk of substance abuse (20). The study results showed that caregivers with a higher education level were less likely to be depressed, which was consistent with studies in Kenya and Tunisia (51, 54). This explains why educated caregivers share the responsibility of following up patients to ensure that they take their mediation and visit health facilities.

The most common related misconception concerns the causes of mental disorders, as the majority agreed that the Ethiopian society still associates the causes of mental disorders with magic, the evil eye, and possession, which complicate the illness and magnify the caregiver burden. “I feel like all the people think that the evil eye is the cause” (IDI 005). The other caregivers themselves believed that the main cause of her mother’s sickness was possession: “My mom had fallen unwell, she was possessed by an evil spirit, and she was treated by a sheikh (a religious faith healer), and then she was diagnosed with schizophrenia. The caregivers believed that the first choice of treatment among many would involve seeking help from a sheikh, holy water who would usually treat a person with religious practices. To be honest with you, we also took him to a sheikh to read [Quran maskerat] over him” (IDI 004).

The results of this study showed that caregivers of patients with schizophrenia spectrum disorders and bipolar and related disorders were 2.8 times and 1.69 times more likely to develop depression, respectively, than caregivers of patients with depression-related disorders. This is because schizophrenia is a chronic, disabling disorder. Treatment is expensive, and the aggressive behavior of the illness also affects physical health (55). Symptoms of bipolar disorder, including violence, aggression, hyperactivity, and disinhibition, are major sources of distress for caregivers (56).

The odds of developing depression were 1.98 times higher among those respondents who gave care for more than 5 years than those who gave care for less than 1 year, which is in line with the study conducted in Ethiopia, Saudi Arabia, and Texas (49, 57, 58). One probable explanation could be that the patient requires constant and ongoing care for extended periods of time, and the caregiver’s stressful role is highly correlated with the patient’s duration of caregiving, trouble finding employment, and financial difficulties or increasing the care cost (50).

This finding suggests that caregivers were 2.20 times more likely to have depression among participants who had poor social support as compared with those who had strong social support, which is in agreement with the previous results in Ethiopia and China (21, 59, 60), possibly because inadequate social support has been associated with sadness and feelings of isolation. It has also been shown to modify brain activity and increase the likelihood of alcohol consumption, cardiovascular diseases, and suicide. Additionally, it provides a safeguard against unhealthy habits and negative health outcomes (17). Inadequate social support was the most powerful predictor of depressive symptom among caregivers of individuals with mental illness (53). This is supported by qualitative findings: “… yes … yes for sure no one is interested to help me because of the nature of the illness no one interested to give support informs of emotional, financial, in one occasion I can’t brought the medication while ask the neighbor no one borrowed me” (IDI 008).

The results of this study showed that the odds of developing depression were 2.33 times higher among participants who had perceived stigma compared with those who did not, which is supported by a study conducted in Ethiopia (21, 61). A possible explanation is that high levels of personal or self-stigma are also correlated with high psychological distress, decreased social functioning, and impaired quality of life (51). This is in agreement with the qualitative findings of the participants: “one scenario what happened most of the society call me you are a father of crazy son so you may be the same to him and they are not interested to involve in social activity and they enforce to restrain him in the home” (IDI 0011).

## Conclusion

The prevalence of depression among caregivers of patients with severe mental illnesses is high. Being female, illiterate, and divorced/widowed, providing care for patients with schizophrenia spectrum disorder and bipolar disorder, giving care for more than 5 years, having chronic medical illness, providing care to severely ill patients, having poor social support, and being stigmatized were significantly associated with depression. Policymakers at all levels should design and implement policies to guarantee the inclusion of caregiver interventions in the mental health system.

### Limitation of the study

The study findings might be prone to response bias due to the patients’ self-reporting, and they do not provide an objective measure of depression. The questionnaire had some sensitive issues that may lead to social desirability.

### Strength of the study

To minimize bias, we used a standardized and pre-tested questionnaire; the response rate in this study was high, which helped to reduce the probability of non-response.

This was an explanatory study that gave detailed insights into participants’ experiences and impacts of caregiving for individuals with severe mental illness.

## Data Availability

The raw data supporting the conclusions of this article will be made available by the authors, without undue reservation.
